# Multi-antigen avian influenza a (H7N9) virus-like particles: particulate characterizations and immunogenicity evaluation in murine and avian models

**DOI:** 10.1186/s12896-016-0321-6

**Published:** 2017-01-07

**Authors:** Che-Ming Jack Hu, Chu-Yang Chien, Ming-Tsan Liu, Zih-Syun Fang, Sui-Yuan Chang, Rong-Huay Juang, Shih-Chung Chang, Hui-Wen Chen

**Affiliations:** 1 Institute of Biomedical Sciences, Academia Sinica, Taipei, Taiwan; 2 Research Center for Nanotechnology and Infectious Diseases, Taipei, Taiwan; 3 Department of Veterinary Medicine, National Taiwan University, Taipei, Taiwan; 4 Center for Research, Diagnostics and Vaccine Development, Centers for Disease Control, Taipei, Taiwan; 5 Department of Clinical Laboratory Sciences and Medical Biotechnology, National Taiwan University College of Medicine, Taipei, Taiwan; 6 Department of Biochemical Science and Technology, National Taiwan University, Taipei, Taiwan

**Keywords:** Avian influenza virus A (H7N9), Virus-like particles, Vaccine

## Abstract

**Background:**

Human infection with avian influenza A virus (H7N9) was first reported in China in March 2013. Since then, hundreds of cases have been confirmed showing severe symptoms with a high mortality rate. The virus was transmitted from avian species to humans and has spread to many neighboring areas, raising serious concerns over its pandemic potential. Towards containing the disease, the goal of this study is to prepare a virus-like particle (VLP) that consists of hemagglutinin (HA), neuraminidase (NA) and matrix protein 1 (M1) derived from the human isolate A/Taiwan/S02076/2013(H7N9) for potential vaccine development.

**Results:**

Full length HA, NA, and M1 protein genes were cloned and expressed using a baculoviral expression system, and the VLPs were generated by co-infecting insect cells with three respective recombinant baculoviruses. Nanoparticle tracking analysis and transmission electron microscopy were applied to verify the VLPs’ structure and antigenicity, and the multiplicity of infection of the recombinant baculoviruses was adjusted to achieve the highest hemagglutination activity. In animal experiments, BALB/c mice and specific-pathogen-free chickens receiving the VLP immunization showed elevated hemagglutination inhibition serum titer and antibodies against NA and M1 proteins. In addition, examination of cellular immunity showed the VLP-immunized mice and chickens exhibited an increased splenic antigen-specific cytokines production.

**Conclusions:**

The H7N9 VLPs possess desirable immunogenicity in vivo and may serve as a candidate for vaccine development against avian influenza A (H7N9) infection.

**Electronic supplementary material:**

The online version of this article (doi:10.1186/s12896-016-0321-6) contains supplementary material, which is available to authorized users.

## Background

Human infection with avian influenza A (H7N9) was first reported in China in March 2013, and since then hundreds of human cases have been confirmed. The disease is associated severe respiratory illness, and the high mortality rate has garnered increasing attention globally [1, 2]. The ability of the virus to transmit from avian species to humans has contributed to its spread to neighbouring areas of China and raises serious concerns over its pandemic potential, particularly in areas with poultry farming industry. With the growing need for countermeasures against the infectious threat, development of effective vaccines is of increasing importance. In addition to ongoing efforts on developing human vaccines against avian influenza virus A/H7N9, formulations that can effectively mount immunity in avian models are of significant public health and economic considerations as they may contain interspecies transmissions and improve overall poultry health. With the aim to prepare a vaccine candidate towards both human and avian applications, we prepared and characterized an A/H7N9-mimicking virus-like particle (VLP). The immune-potentiating effect of the VLP was evaluated in both a mammalian (mouse) and an avian (chicken) model to examine the particles’ potential as an anti-viral vaccine.

Similar to other influenza A viruses, H7N9 viral capsids are comprised of three primary proteins, including hemagglutinin (HA), neuraminidase (NA), and matrix protein 1 (M1). Upon co-expression, these three proteins self-assemble into nanoparticles, which are responsible for packaging viral genomes for disease transmission [3]. Previously, VLPs, which are non-infectious particles devoid of any viral genes, have been shown to induce protective immunity against influenza and other viral diseases [4–9]. The VLPs’ morphological and antigenic semblance to native virions facilitate effective immune processing, making them a compelling alternative to free viral antigens as vaccine candidates. In the present study, VLPs consisting of hemagglutinin (HA), neuraminidase (NA) and matrix protein 1 (M1) derived from the human isolate A/Taiwan/S02076/2013 (H7N9) were prepared for vaccine development. A combinatorial baculoviral system was applied to generate VLPs co-expressing the three viral proteins, which have been recognized as major antigenic targets of the H7N9 virus [10, 11]. The resulting VLPs were assayed for their hemagglutination activity. Nanoparticle tracking analysis was applied to verify the size, surface charge, and concentration of the VLPs, and transmission electron microscopy following immunogold staining was performed to validate the VLPs’ multi-antigenic nature. A mouse model was first used to validate the VLPs’ immunogenicity, and a particular emphasis was placed on the humoral and cellular immune responses in a chicken model following vaccination with either VLPs or free protein antigens. Results observed from the study offer hope that the formulation may find applications in both clinical and agricultural settings.

## Methods

### Cells and recombinant baculoviruses

*S. frugiperda* Sf9 (ATCC CRL-1711) and Sf21 (Invitrogen, Carlsbad, CA) insect cells were maintained as suspension cultures in Grace’s medium (Invitrogen, Carlsbad, CA) and supplemented with 10% FBS (Thermo Scientific, Rockford, IL) and 1% P/S/A antibiotics (Biological Industries, Beit-Haemek, Israel).

Recombinant baculoviruses were prepared by using the Bac-to-Bac baculovirus expression system (Invitrogen). Briefly, three separate recombinant plasmids were constructed by inserting full HA, NA, and M1 genes of A/Taiwan/S02076/2013(H7N9) (accession no. KF018045, KF018047, and KF018048) into the pFastBac-1 vector using primers listed in Table 1. The recombinant pFastBac-1 shuttle vectors were then transposed to the bacmid in *E. coli* strain DH10Bac, and recombinant bacmid was purified using the HiPure Plasmid Midiprep kit (Invitrogen). Sf9 cells were used for transfection with the recombinant bacmid. Right before transfection, 8 μl of the Cellfectin II Reagent (Invitrogen) was diluted in 100 μl of Grace’s medium (without antibiotics and serum), and 1 μg of bacmid DNA was diluted in 100 μl of Grace’s medium (without antibiotics and serum). Subsequently, the diluted bacmid DNA and diluted Cellfectin II were combined and incubated for 30 min at room temperature. The DNA-lipid mixture was then added onto the cells dropwise and incubated at 27 °C for 4 h. Following removal of transfection mixture, fresh cell medium was added to the cells. After 72 h, recombinant baculoviruses were harvested from the supernatant and designated as rBac-H7, rBac-N9, and rBac-M1, respectively. The recombinant baculoviruses were subsequently amplified in Sf9 cells, and virus titers were determined by plaque assays in Sf21 cells.

**Table 1:** Primer sets used in the plasmid construction

Primer	Target	^a^Sequence	Sense	Product
HA-SpeI-F	H7/HA	GCCTAG**ACTAGT**ATGAACACTCAAATCCTG	+	1683 bp
HA-KpnI-R	H7/HA	GCAC**GGTACC**TTATATACAAATAGTGCACC	-	
NA-BamHI-F	N9/NA	TAGATC**GGATCC**ATGAATCCAAATCAGAAG	+	1398 bp
NA-KpnI-R	N9/NA	GATC**GGTACC**TTAGAGGAAGTACTCTATTT	-	
M1-BamHI-F	M1	TAGATC**GGATCC**ATGAGTCTTCTAACCGAG	+	759 bp
M1-HindIII-R	M1	AGCT**AAGCTT**TCACTTGAACCGCTGCAGTT	-	

^a^The restriction sites are bold

### Immunofluorescence assay (IFA)

Sf9 cells were infected with rBac-H7, rBac-N9, and rBac-M1 at a multiplicity of infection (MOI) of 2. Three days later, the monolayer of cells was washed and fixed with 80% acetone at −20 °C for 20 min. Rabbit polyclonal antibody against H7/HA peptide (Sino Biological, Beijing, China), rabbit polyclonal antibody against N9/NA peptide (ProSci, Poway, CA), and mouse polyclonal antibody against a recombinant M1 protein were applied at 1:10,000, 1:2,000, and 1:1,000 dilutions respectively and incubated for 1 h. After PBS wash, cells were further incubated with FITC-conjugated anti-rabbit or anti-mouse IgG (Jackson ImmunoResearch Laboratories, West Grove, PA). Fluorescence signal was observed under a fluorescence microscope.

### Production and purification of H7N9 recombinant proteins and VLPs

H7, N9 and M1 recombinant proteins were harvested from lysed Sf9 cells that were infected with rBac-H7, rBac-N9, or rBac-M1 (MOI = 2). After 72 h, the cells were washed and lysed with the I-PER insect cell protein extraction reagent (Thermo Scientific). Recombinant proteins were purified using the Glycoprotein Isolation Kit, ConA (Thermo Scientific) according to the manufacturer’s instructions.

H7N9 VLP was harvested and purified from the culture supernatant of Sf9 cells co-infected with rBac-H7, rBac-N9, or rBac-M1. Briefly, 72 h after co-infection, the cell culture supernatant was centrifuged at 3,000 × g for 20 min, and the VLPs were pelleted by centrifugation at 70,000 × g for 2 h at 4 °C. The pellet was resuspended in TEN buffer (10 mM Tris-base, 1 mM EDTA, and 100 mM NaCl). The resultant solution was layered onto a sucrose gradient solution (20–50% in TEN buffer) and centrifuged at 100,000 × g for 2 h. Particles from each gradient fraction were separately collected for the HA test and TEM analysis (described below), and the fractions demonstrating the highest HA activity and appropriate morphology were pooled as purified VLPs.

The protein concentration was quantified by the Bradford protein assay (Bio-Rad, Richmond, CA) according to the manufacturer’s recommendations. H7N9 recombinant proteins and VLPs were analyzed by 10% SDS-PAGE, and probed with anti-H7/HA, anti-N9/NA, and anti-M1 antibodies in the IFA section. To detect the protein signals, the membranes were incubated with HRP-conjugated anti-rabbit or anti-mouse IgG (Jackson ImmunoResearch Laboratories) at a 1:2,000 dilution for another hour and developed using ECL Western blotting detection kit (Bio-rad).

### Immunogold labeling of VLPs

Purified VLPs were absorbed onto a plasma-discharged copper grid for 2 min and fixed with 4% paraformaldehyde for 5 min. After PBS washes and blocking with 1% BSA (Sigma, St. Louis, MO), the grid was incubated with either anti-H7/HA or N9/NA antibodies (1:200) for 1 h followed by incubation with 6 nm gold-conjugated goat anti-rabbit antibodies (1:20) (Jackson ImmunoResearch Laboratories). After PBS washes, 2% phosphotungstic acid was applied for negative staining. Particles were observed under a transmission electron microscope (TEM) (JEOL JEM-1400).

### Nanoparticle tracking analysis

Particle concentration, size distribution, and surface zeta potential of the expressed H7N9 VLPs were measured by nanoparticle tracking analysis using NanoSight NS-500 (Malvern Instruments Inc., UK) based on the manufacturer’s instructions. The purified influenza virus A/PuertoRico/8/34(H1N1) [12] was included as a reference.

### Optimization of co-infection of recombinant baculoviruses

Four different conditions of co-infection were tested. rBac-HA (at an MOI of 2, 3.6, 4, 5), rBac-NA (at an MOI of 2, 5, 5, 6), and rBac-M1 (at an MOI of 2, 5, 10, 20) were combined respectively and used for co-infection in Sf9 cells. After 72 h, the cell culture supernatant was centrifuged at 3,000 × g for 20 min, and the VLPs were pelleted by centrifugation at 70,000 × g for 2 h at 4 °C. The pellet was resuspended in TEN buffer, and the resultant solution was layered using a sucrose gradient solution (20–50% in TEN buffer) via centrifugation at 100,000 × g for 2 h. Particles from each gradient fraction were separately collected for a hemagglutination test (described below).

### Mice and chickens immunization

Female 6-week-old BALB/c mice were purchased from BioLASCO (Taipei, Taiwan). Two-week-old specific-pathogen-free (SPF) chickens were obtained from JD-SPF Biotech (Miaoli, Taiwan). Animals were randomly divided into different experimental groups (*n* = 6 per group), receiving H7N9 VLPs, HA/NA/M1 free proteins (only for chickens), or PBS. Briefly, 10 μg of VLPs or pre-mixed HA/NA/M1 proteins at a 1:1:1 ratio were emulsified with the complete Freund’s adjuvant and used for the primary immunization using an intramuscular route. For the booster dose, the same amount of the antigen was mixed with the incomplete Freund’s adjuvant. Mouse serum was collected before immunization and 14 days post-immunization (dpi), and mice were sacrificed at 28 dpi. Chicken serum was collected before immunization and at 14, 19, and 26 dpi, and all the chickens were sacrificed at 40 dpi. Mice and chickens were sacrificed by CO_2_ inhalation.

### Hemagglutination (HA) test and hemagglutination inhibition (HI) test

H7N9 VLPs and inactivated H7N9 virions were used as HA antigens in this study. Inactivated H7N9 virions were prepared as previously described [13]. HA and HI tests were performed with standard protocols provided by WHO [14]. Briefly, the HA activity of purified H7N9 VLPs or inactivated H7N9 virions was tested against red blood cells (RBCs), and HA titers were recorded as the highest dilution exhibiting complete hemagglutination. For the HI test, the receptor-destroying enzyme was used for treating animal sera, and HI titers were recorded as the highest serum dilution exhibiting complete hemagglutination inhibition.

### Antigen-specific cytokine expression analysis

Mice spleens were harvested at 28 dpi, and splenocytes were isolated for intracellular cytokine staining assays. Briefly, spleens were minced and passed through 70-μm cell strainers (Corning) to obtain single-cell suspensions. RBCs were lysed using an RBC lysis buffer (eBiosciences), and cells were resuspended in RPMI 1640 medium (Gibco, Grand Island, NY) containing 10% FBS. Viable cells were determined by trypan blue staining. 10^6^ splenocytes were plated in 96-well U-bottom plates (Corning) and were either mock-stimulated or stimulated with 10 μg of H7N9 VLPs in the presence of brefeldin A (GolgiPlug, BD Biosciences, San Diego, CA) for 5 h at 37 °C. Cells were then washed, incubated with 2.4G2 antibody, and labeled with anti-CD3e-FITC, anti-CD4-PerCP-Cy5.5, and anti-CD8a-PerCP-Cy5.5. The cells were then fixed and permeabilized using a Cytofix/Cytoperm Kit (BD Biosciences) and stained with anti-IL4-PE, anti-IFNγ-APC and anti-TNF-PE. All antibodies for mouse experiments were purchased from BD Biosciences. Samples were read on the BD FACSCalibur and analyzed using FlowJo software (Tree Star, CA).

Chicken spleens were harvested at 40 dpi, and splenocytes were isolated and stimulated with H7N9 VLPs in the presence of brefeldin A as described above. For the flow cytometric analysis, cells were washed and labeled with anti-chicken CD4 or CD8 antibodies (AbD Serotec, Raleigh, NC). The amount of CD4+ or CD8+ T cells gated on 2 x 10^4^ lymphocytes was determined. For the quantification of cytokine expression, the stimulated splenocytes were lysed, and total RNA was isolated by TriSolution reagent (GeneMark, Taipei, Taiwan). Real-time reverse transcription polymerase chain reaction (RT-PCR) was performed using iScript (Bio-rad) and iQ SYBR Green Supermix Kit (Bio-rad) with previously described primers for chicken IL-4, IFN-γ and GAPDH [15]. Melting curve analysis following real-time PCR was conducted to verify the specificity for each primer set. All obtained Ct values were normalized to GAPDH. The relative expression of chicken IL-4 and IFN-γ (fold change of PBS-vaccinated control) was determined by a 2^-ΔΔCt^ method [16].

### Enzyme-linked immunosorbent assay (ELISA)

An amount of 100 ng purified NA or M1 protein was coated onto the flat-bottomed 96 well microplate (Nunc, Roskilde, Denmark) overnight at room temperature. After blocking with 5% skim milk (BD Difco, Sparks, MD), the test chicken sera were serially diluted and incubated for 1 h. Following washes, 100 μl of peroxidase-conjugated goat anti-chicken IgY (H + L) (Jackson ImmunoResearch Laboratories) diluted 1:2,000 in blocking buffer was dispensed into each well and incubated for another 1 h. After additional three washes, the wells were incubated with 100 μl of SureBlue Reserve TMB Microwell Peroxidase Substrate (KPL, Gaithersburg, MD), and color was allowed to develop in the dark for 10 min. The reaction was stopped by the addition of 100 μl of TMB stop solution (KPL). All the incubation steps were performed at room temperature. The optical density (OD) at 450 nm was read using an automated plate reader (Thermo Scientific).

### Statistical analyses

Data were analyzed by unpaired t tests or ANOVA followed by Dunnett’s multiple comparison tests using GraphPad Prism (GraphPad Software, San Diego, CA). The *p* values smaller than 0.05 were considered significant.

## Results

### Production of H7N9 VLPs

**Fig. 1:**
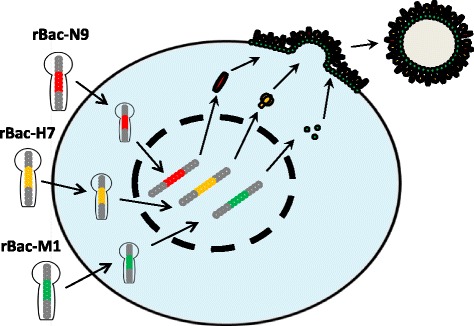
A schematic diagram of H7N9 VLP expression. The genes HA, NA, and M1 were cloned into three recombinant baculoviruses, respectively. Sf9 cells were co-infected by the three recombinant baculoviruses, and the VLPs that assembled HA, NA, and M1 were harvested in the Sf9 culture supernatant

**Fig. 2:**
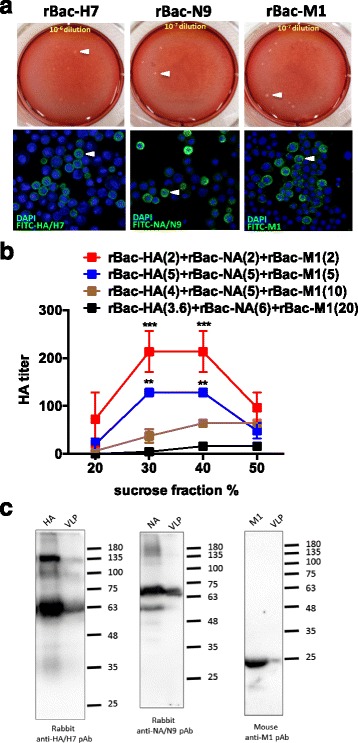
Expression and characterization of H7N9 VLP. **a** The viral titers of three recombinant baculoviruses were determined by plaque assay. The titer of recombinant baculovirus-HA (rBac-HA) was approxmiately 6 × 10^6^ PFU/ml (*left*), the rBac-NA 3 × 10^7^ PFU/ml (center), and the rBac-M1 1.1 × 10^8^ PFU/ml (*right*). The protein expression of HA, NA, and M1 was identified respectively by immunofluorescence assay (lower panel). **b** Sf9 cells were co-infected with rBac-HA, rBac-NA, and rBac-M1 under various MOI combinations, and the VLPs were purified via sucrose gradient centrifugation. Formulation derived from the combinations of rBac-HA (MOI 2), rBac-NA (MOI 2), and rBac-M1 (MOI 2) co-infection exhibited the highest HA activity. **c** H7N9 VLP, H7/HA protein, N9/NA protein, and M1 proteins were detected by Western blot

The full length HA (1,683 nt), NA (1,398 nt), and M1 (759 nt) genes of A/Taiwan/S02076/2013(H7N9) were cloned into three separate recombinant baculoviruses. As indicated in Fig. 1, Sf9 cells were co-infected by the three recombinant baculoviruses, rBac-H7, rBac-N9, rBac-M1, to generate H7N9 VLPs. The titer of each recombinant baculovirus was determined as 6 × 10^6^ plaque-forming unit (PFU)/mL, 3 × 10^7^ PFU/mL, and 1 × 10^8^ PFU/mL, respectively, by plaque assays (Fig. 2a, upper panel). The expression of HA, NA, and M1 proteins in Sf9 cells was identified by IFA using FITC-labelled antigen-specific antibodies. In Sf9 cells nuclei-stained with DAPI, FITC signal was observed in the cytoplasm (Fig. 2a, lower panel), indicating successful protein expression by the respective recombinant baculovirus. In contrast, no FITC signal was observed from the uninfected Sf9 cells following staining by any of the antigen-specific antibodies (data not shown).

As HA is a primary protein that determines the antigenic signature and virulence of influenza viruses, we applied an HA activity assay using chicken red blood cells to optimize the co-infection condition and the VLP collection protocol. The three recombinant baculoviruses of different combinations of multiplicity of infection were prepared to co-infect Sf9 cells. The culture supernatants of the resulting co-infected Sf9 cells were subsequently collected and purified using sucrose gradient centrifugation. Among four different combinations of MOI, the highest HA titer (1: 2^8^) was obtained from the co-infection condition at an MOI of 2 for each of the recombinant baculovirus. This heightened HA activity was particularly pronounced in fractions containing 30–40 wt.% of sucrose (Fig. 2b). Based on the observation, all VLPs in the remainder of the study were prepared using Sf9 cells co-infected with the three recombinant baculoviruses with an MOI of 2. Plaque assays were applied to ensure undetectable titer of residual baculovirus for every batch of the VLPs.

### Characterization of H7N9 VLPs

Upon collection of purified VLPs, Western blotting analysis was applied to confirm the presence of all three of the HA (66 kDa), NA (68 kDa), and M1 (25 kDa) proteins. Each protein on the VLPs was observed to be largely identical in size to the protein from cells infected individually by rBac-H7, rBac-N9, and rBac-M1 (Fig. 2c). This result indicates the three proteins self-assemble without undergoing further modifications.

**Fig. 3:**
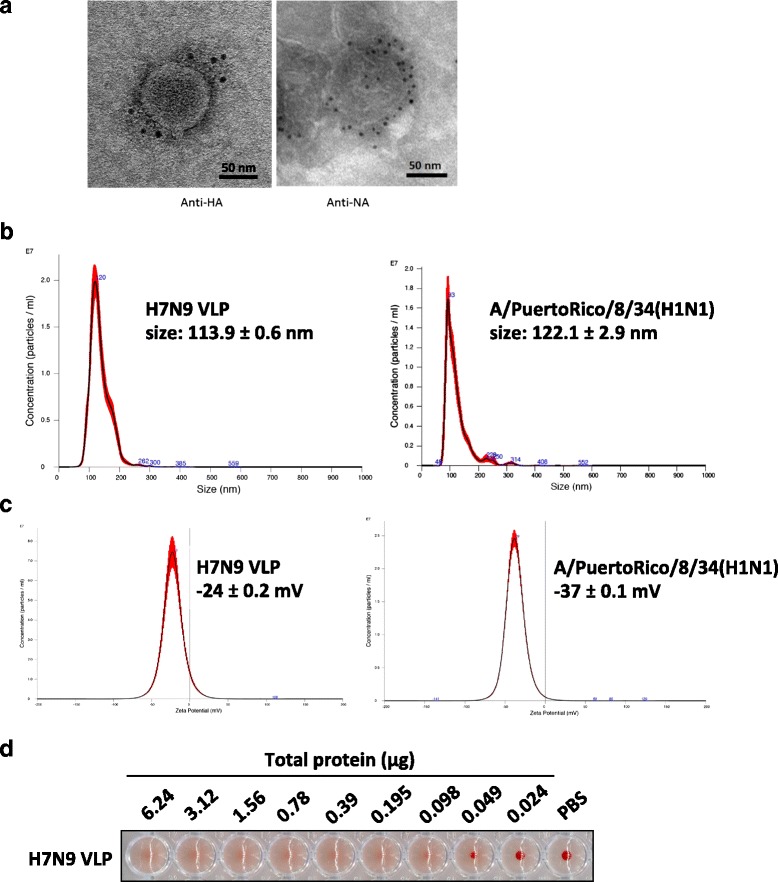
The formation of VLP and proper antigen display of viral proteins were verified. **a** The HA (*left*) and NA (*right*) proteins were detected by antibodies labeled with 6 nm gold under a transmission electron microscope. Scale bars = 50 nm. **b** Size distribution and (**c**) surface zeta potential of the VLPs were analyzed using nanoparticle tracking analysis. Influenza virus A/PuertoRico/8/34(H1N1) was analyzed under the same settings as a reference. **d** Hemagglutination activity of H7N9 VLPs was assessed by a hemagglutination assay. The total protein contents of H7N9 VLP are indicated based on a two-fold serial dilution. PBS was included as the negative control

To examine the formation and antigen display of the H7N9 VLPs, immunogold staining was performed. Under the TEM visualization, particles approximately 120 nm in diameter were observed, confirming successful preparation of VLPs that resemble the morphology of H7N9 viruses. Labeling by immunogold further confirmed the presence of HA proteins (Fig. 3a, left) and NA proteins (Fig. 3a, right) on the surface of the particles. Examination by nanoparticle tracking analysis revealed a unimodal particle size distribution for the VLPs with an average particle diameter of 113.9 ± 0.6 nm, which is similar to that of a native H1N1 virus (122.1 ± 2.9 nm) observed using the technique (Fig. 3b). The surface charge of the VLPs is −24 ± 0.2 mV, which is also similar to that measured from the H1N1 virus (−37 ± 0.1 mV) (Fig. 3c). In addition, the nanoparticle tracking analysis provided insight to the mass of each individual VLP. Based on the observed particle concentration and protein quantification by Bradford assay, it was approximated that there are 1.11 × 10^9^ particles per 1 μg of the VLPs, yielding a molecular weight of roughly 5.42 × 10^8^ Da per particle. Protein quantification showed that the HA protein contributed to approximately 10% of total proteins in collected H7N9 VLPs (Additional file 1: Figure S1), translating to ~800 HA proteins per VLP. The number of HA proteins per VLP is in line with the estimated number in native virion [17]. The purified H7N9 VLPs agglutinate RBCs at a minimum total protein amount of 0.098 μg (Fig. 3d), indicating proper display of the receptor-binding domain of HA protein in the VLPs.

### H7N9 VLP immunization elicited hemagglutination-inhibition antibody response in mice

**Fig. 4:**
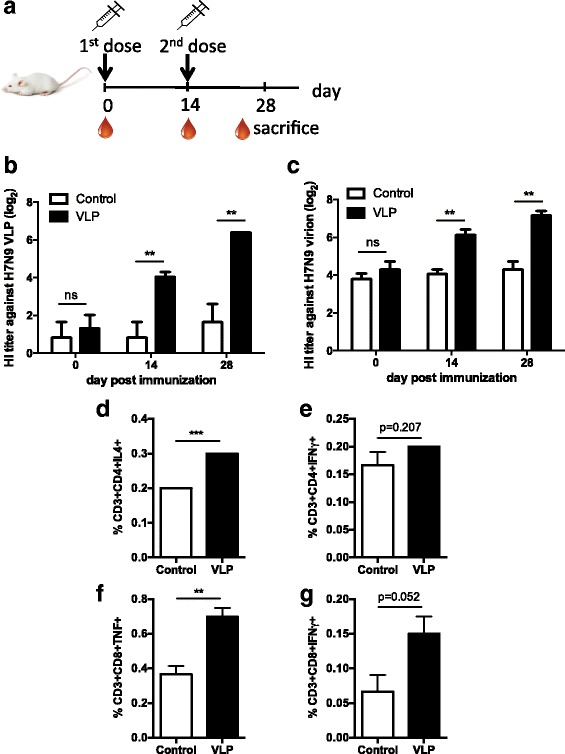
The immunogenicity of H7N9 VLP in a mouse model. **a** Mice (*n* = 6 per group) were immunized with VLPs (10 μg per mouse intramuscularly on day 0 and 14) or PBS, and blood samples were collected. Mice receiving H7N9 VLPs showed significantly increased serum HI titer against the H7N9 inactivated virions (**b**) or H7N9 VLPs (**c**). Mice splenocytes were isolated on day 28 and restimulated ex vivo with the H7N9 VLPs*.* The antigen-specific cytokine responses were detected by intracellular cytokine staining. Comparing with the PBS control, mice receiving the H7N9 VLPs showed elevated CD4 IL-4 and CD8 TNF production (**d**, **f**), whereas the production of CD4 IFN-γ or CD8 IFN-γ revealed no statistical difference (**e**, **g**). Error bars are mean ± SEM. *p < 0.05, **p < 0.01, and ***p < 0.001

To validate the VLPs’ immunogenicity, mice were immunized twice with 10 μg of the VLP (approximately 1 μg of HA content) with a two-week interval (Fig. 4a). Blood was collected right before each vaccination for analysis. The level of serum HI antibody was evaluated by an HI test. As compared to the PBS-immunization control group, VLP-immunized mice exhibited a higher HI antibody response after a primary and a booster vaccination. On day 28, the average HI antibody endpoint dilution titer reached 1:80 and 1:160 against 4 hemagglutination units of H7N9 VLPs and inactivated H7N9 virions respectively (Fig. 4b, c). The study validates the H7N9 VLP’s ability to elicit an anti-HA humoral response in mice.

### H7N9 VLP immunization promotes virus-specific T cell immunity in mice

To examine splenic virus-specific T cell response in H7N9 VLP-immunized mice, intracellular cytokine staining was conducted on the harvested splenocytes on the day of sacrifice. In VLP-vaccinated mice, production of IL-4 and TNF were shown to be elevated in splenic CD4 and CD8 T cells respectively (Fig. 4d and f). Although statistical significance was not reached, the mean percentage of IFN-γ-producing CD4 and CD8 T cells in mice spleens was observed to be greater than the control group following VLP vaccination (Fig. 4e and g). The results suggest that the VLP is capable to raising viral antigen-specific T cell immunity in mammals.

### H7N9 VLP immunization induced HI, anti-NA, and anti-M1 humoral responses in chickens

**Fig. 5:**
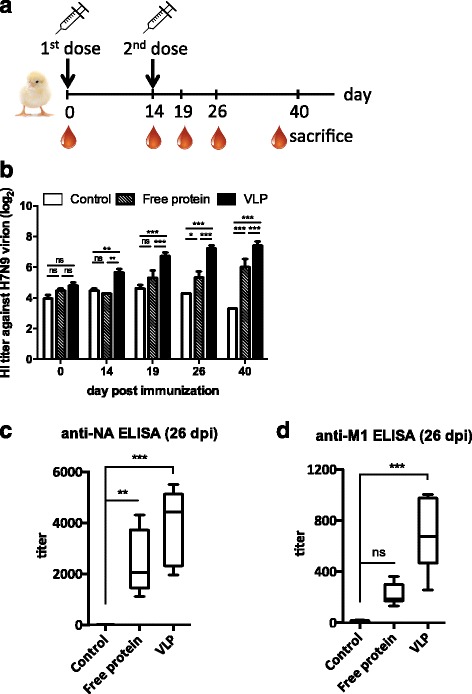
The immunogenicity of H7N9 VLP in a chicken model. **a** SPF chickens (*n* = 6 per group) were immunized with VLPs, HA/NA/M1 free proteins (10 μg per chicken intramuscularly on day 0 and 14) or PBS, and blood samples were collected. **b** Chickens receiving H7N9 VLPs showed significantly increased serum HI titer against the H7N9 inactivated virions. Error bars are mean ± SEM. **c**, **d** On day 26 post-immunization, chickens receiving VLPs exhibited higher serum ELISA titers against purified NA and M1 proteins as compared to the PBS control. Lines and boxes represent upper extreme, 25th, 50th, 75th percentile, and lower extreme. *p < 0.05, **p < 0.01, and ***p < 0.001

To examine the immune-potentiating effect of the H7N9 VLPs in avian species, two-week-old SPF chickens were administered with a primary and a booster vaccination of 10 μg of VLPs on day 0 and day 14 (Fig. 5a). To further evaluate the VLPs’ effectiveness, an additional group of chickens were inoculated with 10 μg of free antigens consisting of HA, NA, and M1 at an 1:1:1 molar ratio for comparison. A group administered with PBS was prepared in parallel as control. Blood was collected on day 0, 14, 19, 26, and 40 to assess the serum HI titers. In experiments using inactivated H7N9 virions as HA antigens, changes in HI serum titers could be observed after day 14 (Fig. 5b). Even though chickens receiving free proteins also showed increased HI titers as compared to the PBS control, the VLP-vaccinated group had significantly higher HI titers. A low HI activity was observed for the PBS control. Over the observation period, HI titers for both VLP and free protein groups increased over time, reaching a peak at 173.33 and 83.33 respectively.

Anti-NA and anti-M1 titers in the serum of the vaccinated chickens were also quantified by ELISA with the blood samples collected on day 26 post immunization. For the anti-NA titer, immunization with free protein was found to elevate the titer to a median value of 2,434, whereas immunization with the VLP resulted in a median titer of 3,957 (Fig. 5c). Similarly, VLP immunization elicited higher anti-M1 titer (median = 685) as compared to free protein immunization (median = 221) (Fig. 5d). The experimental results confirm the presence of HA, NA, and M1 on the VLPs. The VLPs’ are also demonstrated to possess superior capability in eliciting antigen-specific humoral responses as compared to free protein antigens.

### H7N9 VLP elicited virus-specific T cell response in chickens

**Fig. 6:**
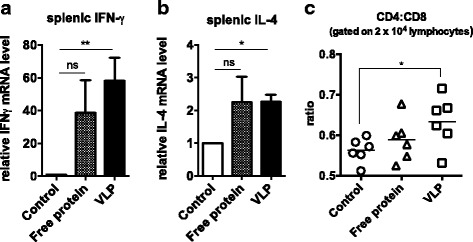
Cell-mediated immunity response induced by H7N9 VLP in chickens (*n* = 6 per group). Splenocytes isolated from chickens on day 40 after immunization were stimulated ex vivo with H7N9 VLPs, and the cell-mediated response was evaluated. **a** Splenic IFN-γ and (**b**) IL-4 mRNA levels were investigated using real-time PCR. By using flow cytometry, chickens receiving VLPs exhibit significantly higher ratio of splenic CD4^+^ / CD8^+^ cell count (**c**), indicating stronger T helper cell response induced by H7N9 VLP vaccination. Error bars are mean ± SEM. *p < 0.05, **p < 0.01, and ***p < 0.001

To analyze antigen-specific T cell responses in chickens, chicken spleens were harvested on day 40 post immunization. Following splenocyte isolation and stimulation with H7N9 VLPs, the splenocytes were lysed and real-time RT-PCR was performed to quantify IFN-γ and IL-4 mRNA expression levels (Fig. 6a and b). It was observed that immunization with free protein formulation and VLPs increased the IFN-γ and IL-4 levels, indicating elicitation of both Th1- and Th2-directed immune responses by both formulations. Upon statistical analysis, however, only the VLPs induced significantly higher IFN-γ and IL-4 expression, whereas enhancement by the free protein formulation did not achieve statistical significance owing to a high degree of variability. The results indicate that the immune responses induced by the free proteins can be highly variable. In contrast, immunization with the VLPs facilitated a more consistent and robust induction in both Th1 and Th2 responses.

CD4/CD8 ratio was also analyzed using flow cytometric analysis (Fig. 6c). On day 40 post immunization, VLP immunization was shown to significantly increase the CD4/CD8 ratio from a mean value of 0.56 to 0.64 (*p* = 0.03). While the immunization with the free protein formulation also increased the mean value of CD4/CD8 ratio, no statistical significant was observed (*p* = 0.31). The results corroborate a more robust and consistent immune response upon exposure to H7N9 virions following VLP immunization.

## Discussion

Since the identification of the avian influenza A (H7N9) infection in human in March 2013, the viral threat has prompted a global effort to develop vaccine candidates as humans have little immunity to this reassorted virus [18–20]. This leap of the virus from birds to humans was attributed to multiple reassortment events, and continuing reassortment events may lead to emergence of more infective strains. Disease management at both the human and animal levels are thus of major importance. In this study, A/Taiwan/S02076/2013(H7N9), a strain isolated in Taiwan [13], was used for the development of a vaccine candidate through the preparation of VLPs. Co-infection of recombinant baculoviruses in insect cells yielded influenza A/H7N9 VLPs that coexpressed HA, NA, and M1 proteins, which have molecular weights consistent with previously published studies [10, 11]. The VLPs are monodisperse and contain approximately 800 HA proteins per particles. These VLPs were found to induce proper humoral and cellular immunity against the H7N9 virus in animal models. In particular, the VLPs were found to be superior in eliciting viral-antigen-specific humoral and cellular immune responses as compared to a free protein formulation in an avian model, which has not been investigated previously.

VLPs are a versatile system that presents a compelling alternative to conventional vaccine formulations as they possess the morphological semblance to natural viral particles. In the present study we applied nanoparticle tracking analysis and transmission electron microscopy to validate the structure and antigenicity of the VLPs. These analyses showed virus-like features consistent with other VLP formulations reported in previous studies [6, 10, 11, 21]. It is worth noting that although an Sf9 insect cell system was adopted for the production of the H7N9 VLPs in this study, the VLP platform is highly versatile and may be produced using other culture systems. For instance, a previous study by Chang et al. employed an alternate High Five insect cell culture system for VLP production [22, 23], demonstrating high production yield upon High Five system optimization. In the present study, we showed that varying the MOI of the different recombinant baculoviruses impacted the VLP production, offering information toward further optimization of the Sf9 system for VLP generation.

We first validated the potency of the VLPs in a mouse model. Given that neutralizing antibodies against the globular head of hemagglutinin protein are the primary mediators of most vaccine-induced protection against influenza, a hemagglutination inhibition assay was used to examine the VLPs as a vaccine candidate against H7N9. An HI antibody titer of 1:40 is the accepted correlate of protection for human HA split inactivated vaccines [24]. Immunization with the VLPs resulted in a mouse serum HI titer of 1:160 against the H7N9 virions. Although future animal studies with viral challenges are warranted, the present result validates the immunopotentiation effect of our VLPs, which is on par with those in previous reports.

Despite previous studies that examined different formulations of H7N9 VLPs in mouse models [9–11, 25, 26], no examination of H7N9 vaccination in avian models has been reported to the best of our knowledge. Given that the management of the avian influenza virus would benefit from both human and poultry vaccinations, we investigated the VLPs’ vaccination effect in SPF chickens. As compared to a free protein formulation, the VLP vaccination yielded increased HI serum titers, anti-NA titers, and anti-M1 titers. It is also worth noting that upon examination of cellular immune responses, we observed that the VLP immunization resulted in elevated splenic IFN-γ and IL-4 upon subsequent viral exposure. The cellular immune responses elicited by VLPs in SPF chickens are consistent with previous studies examining VLP immunizations in other animal models [21].

In the present study, we purified our VLPs and made sure that no detectable baculovirus titer was present in any of our batches. The purification is critical as residual baculoviral proteins were previously shown to trigger innate immune responses through TLR9 and other pattern recognition receptors [27, 28]. Towards future clinical translation, the elimination of residual baculovirus contamination is of high importance.

Even though the free protein formulation, which is a mixture consisting of free HA, NA, and M1 proteins at a 1:1:1 ratio, also elicited humoral and cellular responses in our study, the responses were either weaker or more variable as compared to those elicited by the VLPs. The finding fortifies the notion that the H7N9 VLPs may serve as a compelling vaccine candidate in poultry farming, which typically employs inactivated or subunit vaccines for disease management [29–31].

## Conclusions

Our study has prepared H7N9 VLPs co-expressing HA, NA, and M1 as a vaccine candidate against avian influenza. The platform elicited more potent humoral immune responses and more consistent cellular immune responses as compared to the free protein formulation. Although future studies involving viral challenges are warranted, the platform presents a viable candidate for avian influenza management in both human and animal settings.

## Additional file

Additional file 1: Figure S1. HA protein quantification of VLP. The equivalent amount of HA protein on the H7N9 VLP. (PDF 331 kb)

## Abbreviations

dpi: Day post-immunization
ELISA: Enzyme-linked immunosorbent assay
HA: Hemagglutinin
HI: Hemagglutination inhibition
IFA: Immunofluorescence assay
M1: Matrix protein 1
MOI: Multiplicity of infection
NA: Neuraminidase
OD: Optical density
PFU: Plaque-forming unit
RBC: Red blood cell
RT-PCR: Reverse transcription polymerase chain reaction
SPF: Specific-pathogen-free
TEM: Transmission electron microscope
VLP: Virus-like particle
