# Supplementary material for: Multi-antigen avian influenza a (H7N9) virus-like particles: particulate characterizations and immunogenicity evaluation in murine and avian models
Source: BMC Biotechnol. 2017 Jan 7;17:2. doi: 10.1186/s12896-016-0321-6 (PMC5219756; doi:10.1186/s12896-016-0321-6)

Fig. S1. Quantification of HA antigens in the H7N9 VLPs. Equal amount (2 μg) of recombinant HA protein (rH7) and purified H7N9 VLPs were analyzed by Western blot. ImageJ software was used for quantification analysis.

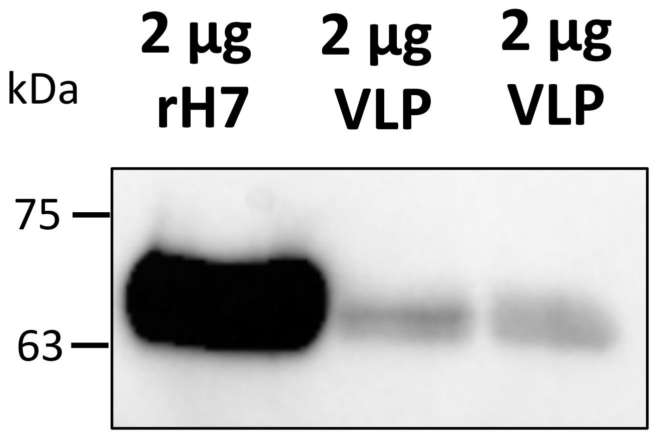

Supplement: Additional file 1: Figure S1. — HA protein quantification of VLP. The equivalent amount of HA protein on the H7N9 VLP. (PDF 331 kb) [file 12896_2016_321_MOESM1_ESM.pdf]
